# Hyperoside attenuates dextran sulfate sodium-induced colitis in mice possibly via activation of the Nrf2 signalling pathway

**DOI:** 10.1186/s12950-017-0172-5

**Published:** 2017-11-14

**Authors:** Lei Yang, Lei Shen, Yue Li, Yanxia Li, Shijie Yu, Shanshan Wang

**Affiliations:** Department of Gastroenterology, Key Laboratory of Hubei Province for Digestive System Disease, Renmin Hospital of Wuhan University, Wuhan, 430060 China

**Keywords:** Hyperoside, DSS-induced colitis, Colonic inflammation, Apoptosis, Nrf2

## Abstract

**Background:**

Hyperoside (Hyp) is a flavonoid glycoside compound that has been demonstrated to have anti-inflammatory, anti-apoptotic and antioxidant effects. However, the impact of Hyp on inflammatory bowel disease (IBD) has not been previously explored. Thus, we evaluated the role of Hyp in dextran sodium sulfate (DSS)-induced acute colitis in mice.

**Methods:**

We established a mouse model of experimental acute colitis by treating mice with drinking water supplemented with 3.0% DSS for 7 days. The disease activity index (DAI), colon length, histological features and colonic malondialdehyde (MDA) levels were examined using appropriate methods, and COX-2 expression was examined by immunohistochemistry. TNF-α, IL-4, IL-6, IL-10, NF-κB p65, Bcl-2, Bax, Caspase-3, nuclear factor-erythroid 2-related factor 2 (Nrf2), hemeoxygenase-1 (HO-1) and superoxide dismutase (SOD) levels in colorectal tissues were detected by RT-PCR and western blotting.

**Results:**

Hyp significantly attenuated DSS-induced changes in the DAI as well as DSS-induced colonic shortening and histological changes. Hyp also inhibited inflammation, a change reflected by decreases in TNF-α, IL-6, COX-2 and NF-κB p65 expression and increases in IL-10 expression. Hyp suppressed increases in the levels of apoptosis-related proteins, such as Caspase-3 and Bax, but upregulated the level of the anti-apoptotic protein Bcl2. In addition, Hyp also exerted antioxidant effects. The MDA content was decreased, and the expression of Nrf2 and its downstream targets HO-1 and SOD were increased by Hyp.

**Conclusions:**

Based on these findings, Hyp possesses the ability to attenuate colitis, possibly by mitigating colonic inflammation and apoptosis via activation of the Nrf2 signaling pathway.

## Background

Ulcerative colitis (UC), one of the subtypes of inflammatory bowel disease (IBD), is a chronic relapsing and non-specific inflammatory disorder of the gastrointestinal tract. The prevalence of UC is increasing, and UC has serious effects on the health and quality of life of affected patients. Despite improvements in our understanding of UC in recent years, the etiology and pathogenesis of the disease remain unclear and may be associated with several factors. UC is thought to be associated with factors such as genetic susceptibility, imbalances between gastrointestinal probiotics and pathogenic bacteria, intestinal mucosa damage, immune dysfunction and other phenomena. Immune dysregulation likely underlies the basic pathogenesis of IBD [1]. The activation of the NF-κB pathway as well as the release of related inflammatory cytokines and proinflammatory mediators, plays an important role in the pathogenesis of UC [2, 3], and increases in the frequency of apoptosis, a process that results in the loss of intestinal epithelial cells, also participate in IBD progression [4]. Furthermore, the roles of oxidative stress and the oxidant/antioxidant balance in UC development have recently received increasing attention [5].

Nuclear factor-erythroid 2-related factor 2 (Nrf2), a redox-sensitive transcription factor, is one of the members of the Cap-N-Collar family of transcription factors. Nfr2 features a leucine zipper structure [6] and plays a key role in the antioxidant response. When cells are exposed to oxidative stress, Nrf2 is released from Kelch-like ECH-associated protein 1 (Keap1), which sequesters Nrf2 in the cytoplasm, and then binds to Maf or Jun to form a heterodimer in the nucleus. The heterodimer combines with the antioxidant response element (ARE) to promote the expression of genes encoding many phase II detoxification and antioxidant enzymes, including hemeoxygenase-1 (HO-1), superoxide dismutase (SOD), and NOQ1, thereby improving the ability of the cell to remove electrophilic and reactive oxygen species (ROS) [7]. A Nrf2 deficiency has been shown to exacerbate colonic injury in a mouse model of experimental colitis [8–10], whereas pharmacological activation of Nrf2 produced protective effects on the colon [10, 11]. The Nrf2 pathway not only upregulates the expression of various antioxidant enzymes but also downregulates a series of inflammatory mediators and attenuates apoptosis [12, 13]. These Nrf2-mediated processes are vital to the ability of the body to resist different types of injury.

Hyperoside (Hyp, a quercetin-3-O-β-D-pyran galactose glucoside) belongs to the flavonol glycoside family and is extracted from *Hypericum*, *Ericaceae*, *Celastraceae* and other species. Hyp exerts a wide variety of pharmacological effects, including anti-inflammatory, antioxidant, antitumor, analgesic, and immunoregulatory effects. Several studies have reported the anti-inflammatory and antioxidant properties of Hyp. As shown in the studies by Li Y et al*.* [14, 15], Hyp inhibits the activation of NF-κB and suppresses the secretion of proinflammatory cytokines, including TNF-α, COX-2 and iNOS. Based on the results from in vivo and in vitro studies, Hyp enhances the expression of genes encoding many phase II detoxifiers and antioxidants by activating Nrf2 [15, 16]. In addition, Li ZL et al. [17] showed that Hyp exerted protective effects on H_2_O_2_-induced human umbilical vein endothelial cell lesions by attenuating endothelial cell apoptosis.

In the present study, we attempted to determine whether Hyp-mediated Nrf2 upregulation exerts anti-inflammatory and anti-apoptotic effects on DSS-induced UC in a C57BL/6 mouse model.

## Methods

### Animals

We obtained 6- to 8-week-old adult male C57BL/6 mice from Beijing HFK Bioscience Co., Ltd. (Beijing, China). All mice were housed in a room with a controlled temperature (21±2°C) and humidity (50±5%) and were maintained on a 12-h light/dark cycle. The mice had access to a standard mouse diet and sterile water ad libitum and were acclimated to the laboratory conditions for 7 days before undergoing any experimental procedures. All experimental procedures were conducted in accordance with the institutional guidelines for the care and use of laboratory animals of Renmin Hospital of Wuhan University, Wuhan, China, and conformed to the regulations outlined in the National Institutes of Health Guide for Care and Use of Laboratory Animals.

### Reagents

Hyp (purity >98%) was obtained from Shanghai Yuanye Biotechnology Co., Ltd. (Shanghai, China). DSS (MW 36,000–50,000 Da) was obtained from MP Biomedical (Aurora, OH, USA). The malondialdehyde (MDA) reagent kit was purchased from the Nanjing Jiancheng Bioengineering Institute (Nanjing, China). The DAB kit was purchased from Beijing Solarbio Science & Technology Co., Ltd. (Beijing, China). Antibodies against COX-2 and Nrf2 were obtained from Santa Cruz Biotechnology Inc. (CA, USA), and antibodies against NF-κB p65, Bcl-2, Bax, and Caspase-3 were obtained from Cell Signaling Technology, Inc. (Beverly, MA, USA). β-Actin and Lamin B antibodies were purchased from Abcam (Cambridge, UK).

### Experimental design

Twenty-eight mice were randomly allocated into the following four groups (7 mice per group): a control group, a DSS model group, and DSS+Hyp (treated with 80 and 120 mg/kg Hyp, respectively) groups. Hyp was suspended in 0.5% carboxymethyl cellulose (CMC). The mice in the control group received normal drinking water and 0.5% CMC by gavage in the morning for 14 days. The mice in the DSS model group received 0.5% CMC by gavage for 7 days prior to 3% DSS (MP Biomedical. 160110) exposure and were then maintained until sacrifice, whereas the mice in the DSS+Hyp (80 and 120 mg/kg, respectively) groups received Hyp suspended in 0.5% CMC. The body weight, stool consistency, and gross bleeding scores of each animal were recorded to calculate the DAI [18]. The criteria with which the DAI was graded are shown in Table 1. On the morning of day 15, all mice were sacrificed, and the total colon length was measured. The dissected colon was washed gently with PBS. A portion of the distal colon was subsequently fixed with 10% buffered formalin for 24 h, and the remaining tissue was immediately frozen in liquid nitrogen and stored at -80°C until use in subsequent experiments.

**Table 1:** The grading rule of disease activity index (DAI)

Score	Weight loss (%)	Stool consistency	Blood in stool
0	None	0 = normal	0 = negative
1	1–5%	2 = loose stool	2 = Hemoccult positive
2	6–10%	4 = diarrhea	4 = gross bleeding
3	11–20%		
4	>20%		

### Histopathology analyses

A portion of the distal colon, which was fixed with 10% buffered formalin for more than 24 h, was mounted in paraffin and stained with hematoxylin and eosin before observation under a light microscope. The following previously described histological scoring system [19] was used to grade the severity of the tissue damage induced by DSS: percent tissue damage: 0=no tissue damage, 1=1–25%, 2=26–50%, 3=51–75% and 4=76–100%; extent of tissue damage: 1=mucosa, 2=mucosa and submucosa and 3=beyond the submucosa; extent of crypt damage: 1=basal 1/3 damaged, 2=basal 2/3 damaged, 3=only the surface epithelium was intact and 4=the entire crypt and epithelium were lost; and degree of inflammation: 1=slight, 2=moderate and 3=severe.

### Immunohistochemistry analyses

A portion of the colon, which was fixed with 10% buffered formalin and then dehydrated in a graded ethanol series, was mounted in paraffin and then sectioned (4 μm). The sections were dewaxed and rehydrated before being incubated with 3% H_2_O_2_. Sections were then incubated with a COX-2 antibody (dilution 1:50) in a humidified chamber overnight at 4°C. Sections were washed and then incubated with the appropriate secondary antibodies and streptavidin-horseradish peroxidase (HRP) for 30 min at room temperature. Each section was treated with freshly prepared diaminobenzidine (DAB) before being counterstained with hematoxylin. Images were obtained and observed under a microscope.

### Malondialdehyde (MDA) analyses

The MDA content was determined with a commercial kit that assesses MDA levels through a process dependent on thiobarbituric acid (TBA) reactivity, according to the manufacturer’s protocol. We added TBA to a protein-free supernatant, which was obtained by centrifuging colonic tissue homogenates with trichloroacetic acid. The TBA was allowed to react with the supernatant for 60 min at 95°C before the mixture was cooled rapidly. The absorbance of the supernatant was assessed at 532 nm using a spectrophotometer.

### Real-time PCR

Total RNA was isolated from the colonic tissue samples using TRIzol reagent (Invitrogen, Carlsbad, CA), according to the manufacturer’s instructions and was verified by determining the absorbance at 260/280 nm using a spectrophotometer. The RNA was then reverse-transcribed into cDNA with reverse transcriptase (GeneCopoeia, Rockville, USA). Real-time PCR (RT-PCR) was conducted with SYBR Green Master Mix (Vazyme, Nanjing, China). β-Actin, a housekeeping gene, was used as a positive control. The sequences of all primers used for the RT-PCR experiments are presented in Table 2.

**Table 2:** Primer sequences for real-time PCR

Name	Primer	Sequence
β- actin	Forward	5’- CACGATGGAGGGGCCGGACTCATC-3’
	Reverse	5’- TAAAGACCTCTATGCCAACACAGT-3’
Nrf2	Forward	5’- GAGCTAGATAGTGCCCCTGG -3’
	Reverse	5’- CAGGACTCACGGGAACTTCT -3’
SOD	Forward	5’- AACCATCCACTTCGAGCAGA -3’
	Reverse	5’- GGTCTCCAACATGCCTCTCT -3’
HO-1	Reverse	5’- CCCTAATGAGCTGCCCTACA -3’
	Forward	5’- TTCATCCCCACTCTCTTCGG -3’
TNF-a	Forward	5’- ACCCTCACACTCACAAACCA -3’
	Reverse	5’- GGCAGAGAGGAGGTTGACTT -3’
IL-4	Forward	5’- TCTCGAATGTACCAGGAGCC-3’
	Reverse	5’- ACCTTGGAAGCCCTACAGAC-3’
IL-6	Forward	5’-GTTGCCTTCTTGGGACTGAT-3’
	Reverse	5’-ATTAAGCCTCCGACTTGTGA-3’
IL-10	Forward	5’- ATAACTGCACCCACTTCCCA-3’
	Reverse	5’- GGGCATCACTTCTACCAGGT-3’
Bcl-2	Forward	5’- TCGCAGAGATGTCCAGTCAG-3’
	Reverse	5’- ATCTCCCTGTTGACGCTCTC-3’
Bax	Forward	5’-TCATGAAGACAGGGGCCTTT -3’
	Reverse	5’-GTCCACGTCAGCAATCATCC -3’
Caspase-3	Forward	5’- CTACAGCACCTGGTTACTATTC-3’
	Reverse	5’- TACAGTTCTTTCGTGAGCAT-3’

### Cytosolic and nuclear protein extraction and western blotting

Total cytosolic and nuclear proteins were extracted from the colonic tissue samples using the appropriate kits, according to the manufacturer’s instructions (Thermo Fisher Scientific Inc., MA, USA). Protein concentrations were determined with a BCA Protein Assay Kit (Beyotime, Shanghai, China), and the proteins were boiled for 5 min. The proteins (40 μg) were then separated by SDS-PAGE before being transferred to polyvinylidene difluoride (PVDF) membranes (Millipore, CA, USA). After blocking with 5% skim milk in TBST for at least 2 h at room temperature, membranes were incubated with primary antibodies against the following proteins overnight at 4°C: NF-κB p65, Bcl-2, Bax, Caspase-3 and Nrf2. LaminB and β-actin were used as loading controls for the nuclear and total or cytoplasmic protein fractions, respectively. The membranes were washed at least three times before incubation with the appropriate HRP-conjugated secondary antibodies. Protein bands were exposed to film and then quantified using ImageJ software (NIH, Bethesda, MD).

### Statistical analyses

The data obtained from the experiments described above were analyzed using SPSS 16.0 software and are presented as means±S.D. One-way analysis of variance (ANOVA) and Studentʼs t tests were used to assess the differences among and between groups, respectively. *P*-values <0.05 were considered statistically significant.

## Results

### Effects of Hyp on the DAI and colon length

**Fig. 1:**
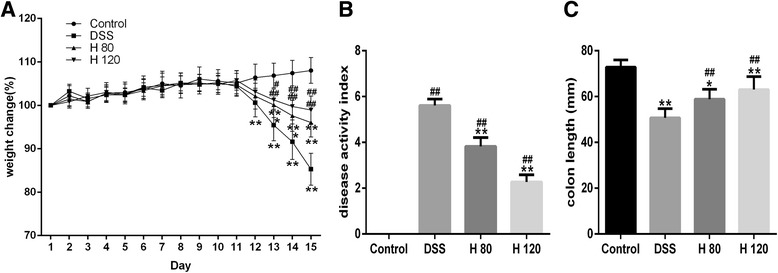
Protective role of Hyp against DSS-induced acute colitis in mice. **a** Body weight changes were measured. **b** Disease activity index (DAI) was detected. **c** Statistics of colorectum length of each group were recorded. H 80 and H 120, mice treated with DSS and Hyp 80 and 120 mg/kg respectively. Data are presented as mean±S.D. (*n* = 7). Significance: *P<0.05, ** *P*<0.01 in comparison with DSS model group; ^#^ *P*<0.05, ^##^ *P*<0.01 in comparison with control group

As shown in Fig. 1b, the DAI (as determined by body weight loss, Fig. 1a; stool consistency and bloody stool scores) was increased in DSS-induced mice compared with healthy control mice in the present study. Hyp-treated mice had a lower DAI than the DSS model group, and the difference in the DAI between the two Hyp-treated groups was statistically significant. As shown in Fig. 1c, the colon length was reduced in DSS-induced mice compared with control mice; however, the Hyp treatment attenuated this effect on the Hyp-treated groups. Thus, Hyp alleviates the symptoms of colitis in the mouse model of DSS-induced colitis.

### Hyp ameliorates histological damage

**Fig. 2:**
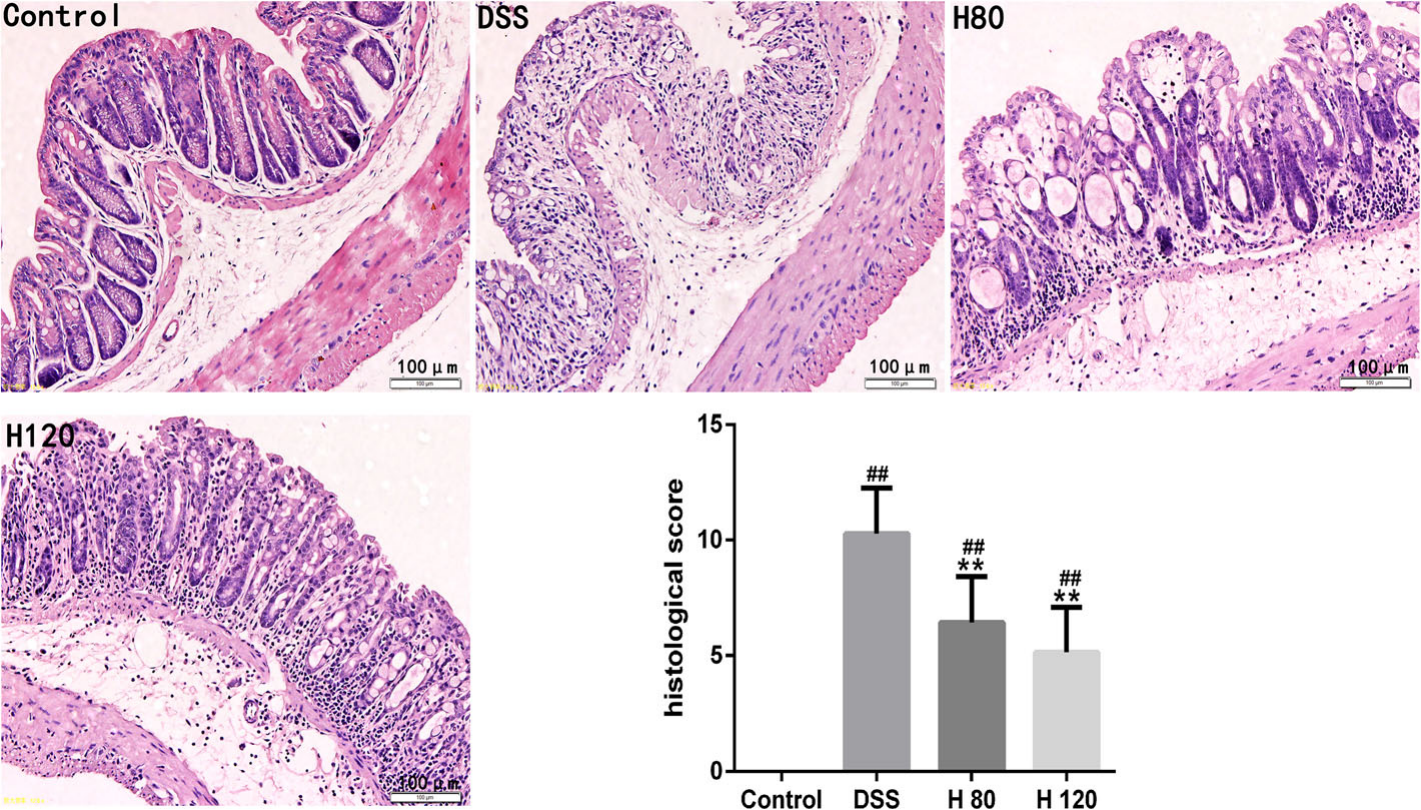
Hyp alleviated DSS-induced colon damage in mice. Representative H&E-stained colorectum sections(magnification 200) and histology score. H 80 and H 120, mice treated with DSS and Hyp 80 and 120 mg/kg respectively. Data are presented as mean±S.D. (*n* = 7). Significance: *P<0.05, ** *P*<0.01 in comparison with DSS model group; ^#^ *P*<0.05, ^##^ *P*<0.01 in comparison with control group

The mice in DSS model group displayed abnormalities in the colonic architecture, such as mucosal ulcerations, glandular defects and inflammatory cell infiltration, whereas the mice in the healthy control group displayed a normal colonic structure. The Hyp treatment ameliorated these histological changes (Fig. 2). Hyp administration also decreased the histological scores in the Hyp-treated groups compared with the DSS model group (Fig. 2), indicating that Hyp exerts significant protective effects on inflammation-induced intestinal damage in mice with DSS-induced colitis.

### Effects of Hyp on colonic inflammation

**Fig. 3:**
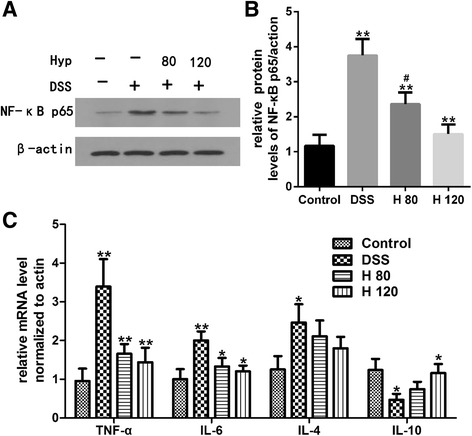
Hyp reduced colonic levels of NF-κB p65,TNF-α, IL-6 and enhanced the levels of IL-10 in DSS-induced mice. **a** The expressions of NF-κB p65 via western blotting. **b** The relative protein expressions of NF-κB p65 were normalized to β- actin. **c** Gene expression of TNF-α, IL-6, IL-4, IL-10 was used to detected via RT-PCR. H 80 and H 120, mice treated with DSS and Hyp 80 and 120 mg/kg respectively. Data are presented as mean±S.D. (n = 7). Significance: *P<0.05, ** *P*<0.01 in comparison with DSS model group. ^#^ *P*<0.05, ^##^ *P*<0.01 in comparison with control group

**Fig. 4:**
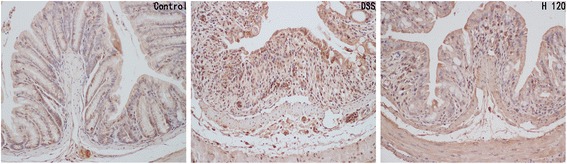
Hyp alleviated the colonic expression of COX2. Immunohistochemical analysis of COX2 (magnification, 200×). H 80 and H 120, mice treated with DSS and Hyp 80 and 120 mg/kg respectively. Data are presented as mean±S.D. (*n* = 7). Significance: *P<0.05, ** *P*<0.01 in comparison with DSS model group; ^#^ *P*<0.05, ^##^ *P*<0.01 in comparison with control group

Inflammation is a major factor during UC progression. Based on the western blotting data, NF-κB p65 was expressed at lower levels in the Hyp-treated groups than in the DSS model group (Fig. 3a and b). Levels of TNF-α, IL-4 and IL-6 mRNA were significantly increased in the DSS model group compared with the healthy control group, indicating that DSS significantly increased intestinal inflammation in the DSS-induced colitis mouse model. However, Hyp decreased the expression of TNF-α and IL-6 mRNA in the colon. However, a significant difference in the expression of IL-4 mRNA was not observed between the DSS- and Hyp-treated groups. IL-10 mRNA was expressed at higher levels in the Hyp-treated groups than in the DSS model group (Fig. 3c). Moreover, COX-2 expression was decreased by treatment with Hyp in the Hyp-treated groups compared with the DSS model group (Fig. 4).

### Effects of Hyp on apoptosis

**Fig. 5:**
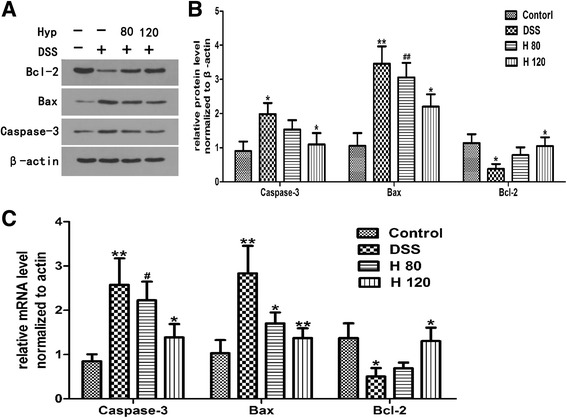
The effects of Hyp on apoptosis. **a** The expressions of Caspase-3, Bax and Bcl-2 via western blotting. **b** The relative protein expressions of Caspase-3, Bax and Bcl-2 were normalized to β- actin. **c** Gene expression of Caspase-3, Bax and Bcl-2 via RT-PCR. H 80 and H 120, mice treated with DSS and Hyp 80 and 120 mg/kg respectively. Data are presented as mean±S.D. (*n* = 7). Significance: *P<0.05, ** *P*<0.01 in comparison with DSS model group; ^#^ *P*<0.05, ^##^ *P*<0.01 in comparison with control group

The expression levels of Caspase-3, Bax and Bcl-2 were quantified by western blotting. Levels of Caspase-3 and Bax protein in the colon were significantly increased in the DSS-induced group compared with the healthy control group, and Bcl-2 protein was expressed at significantly lower levels in DSS-induced mice than in healthy control mice (Fig. 5a and b), indicating that the rate of colonic apoptosis in mice with DSS-induced colitis was increased compared with healthy control mice. Hyp increased the protein expression of Bcl-2 and decreased the protein expression of Caspase-3 and Bax in the Hyp-treated group compared with the DSS model group (Fig. 5a and b). Changes in the levels of Bcl-2, Bax and Caspase-3 mRNA, quantified by RT-PCR, were consistent with changes in the protein levels of Bcl-2, Bax and Caspase-3 (Fig. 5c).

### Hyp decreases the colonic MDA content

**Fig. 6:**
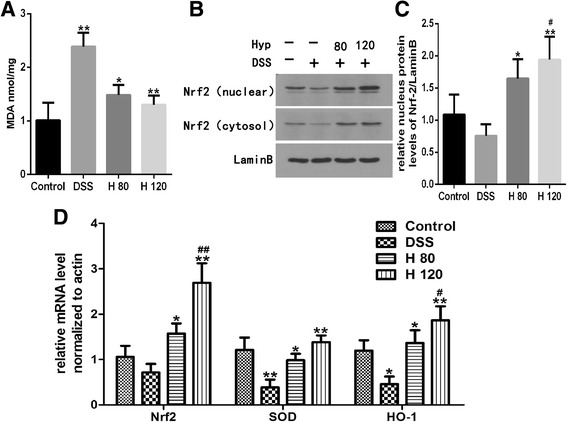
Hyp reduced colonic levels of MDA and activated Nrf2 pathway. **a** The levels of MDA. **b** The expressions of Nrf2 via western blotting. **c** The relative protein expressions of nucleus Nrf2 were normalized to Lamin B. **d** Gene expression of Nrf2、SOD and HO-1 via RT-PCR. H 80 and H 120, mice treated with DSS and Hyp 80 and 120 mg/kg respectively. Data are presented as mean±S.D. (*n* = 7). Significance: *P<0.05, ** *P*<0.01 in comparison with DSS model group; ^#^ *P*<0.05, ^##^ *P*<0.01 in comparison with control group

MDA, an important byproduct of lipid peroxidation, is an important index of oxidation reactions in the cell. The MDA content was significantly increased in the DSS model group compared with the healthy control group. However, the MDA content was dramatically decreased in the Hyp-treated groups compared with the DSS model group (Fig. 6a).

### Effects of Hyp on the Nrf2 signaling pathway

The cytosolic and nuclear levels of the Nrf2 protein were significantly reduced in DSS-induced mice compared with healthy control mice (Fig. 6b and c). Levels of Nrf2, HO-1 and SOD mRNA were detected using RT-PCR, and the levels of HO-1 and SOD mRNA were significantly lower in the colons of DSS-induced mice than in healthy control mice. The Hyp treatment markedly increased the levels of Nrf2, HO-1 and SOD mRNA in DSS-induced mice compared with healthy control mice (Fig. 6d). Based on these findings, Hyp exerted its beneficial effects on DSS-induced colitis through the Nrf2 signaling pathway.

## Discussion

UC is a chronic relapsing intestinal inflammatory disease that is detrimental to the physical and mental health of affected patients and likely increases the risk of colorectal cancer [20]. The drugs that are currently used to alleviate the clinical symptoms and inflammation associated with UC include aminosalicylates, corticosteroids, immunosuppressors and biological agents. However, these drugs also have some adverse effects. Therefore, studies seeking to develop safe, effective and targeted therapies for UC are needed. The present study explored the protective effects of Hyp on DSS-induced UC.

The mouse model of DSS-induced colitis is a well-established experimental model that shares many signs and symptoms with human UC, including weight loss, diarrhea, emaciation, melena, mucosal ulcerations, colonic shortening and inflammatory cell infiltration [3]. Severe diarrhea, bloody stools, weight loss, and decreases in activity and appetite appeared on the third day after DSS exposure. After the experiment, the DSS-induced group exhibited a remarkably higher DAI score than the healthy control group. We observed DSS-induced pathological changes in the colonic tissue samples by HE staining and found that the mice in the DSS model group displayed prominent abnormalities in the colonic architecture, such as mucosal ulcerations, glandular defects and inflammatory cell infiltration. Moreover, the pathological scores were significantly higher in the DSS model group than in the healthy control group. Thus, the UC model was successfully established. Based on our results, Hyp alleviates the symptoms and pathological changes in the intestine that are characteristic of experimental UC in mice.

Natural herbal compounds containing Hyp have been proposed to be safer than conventional medicines and are used widely [21]. The administration of 50-200 mg/kg Hyp exerted a potent hepatoprotective effect on a mouse model of liver injury by upregulating the Nrf2 pathway [15, 22]. In addition, Hyp showed certain toxicity during embryonic/fetal development in rats at the dose of 1000 mg/kg [23]. Based on these findings, our dosing level was safe and efficient. Hyp exerted protective effects on DSS-induced colitis in the present study. However, in other studies, the administration of 10–40 mg/kg Hyp significantly suppressed the NF-κB signaling pathway in mouse models of allergic airway inflammation and pancreatic tumors [14, 24]. The effective range of Hyp concentrations seems to be quite different. The discrepancies in the results may be explained by the use of different disease models, the biological and/or inter-laboratory variability or by a bell-shaped dose-response curve. Therefore, additional pharmacokinetic and toxicology experiments should be performed with Hyp to further elucidate the mechanism of Hyp before its clinical application.

Inflammatory cells and the secretion of related inflammatory mediators play critical roles in the development and progression of UC [25]. NF-κB, a key factor in the immune system, regulates the expression of several proinflammatory factors, such as TNF-α, IL-4, IL-6, COX-2, adhesion molecule-1 and chemokines. These factors eventually induce local inflammation and immune dysfunction, changes that trigger a positive feedback loop leading to the development of and increases in inflammation, resulting in intestinal mucosal damage [26]. Those inflammatory mediators are significantly upregulated not only in experimental colitis [25, 27] but also in patients with IBD [28, 29]. Based on accumulating evidence, the inhibition of inflammatory mediators attenuates experimental models of IBD [14, 30]. IL-10 is an anti-inflammatory factor that plays a protective role in UC. IL-10- and IL10 receptor-deficient mice spontaneously develop colitis and are widely used murine models of IBD [31, 32]. We measured the expression levels of related inflammatory mediators to explore the protective effects of Hyp on colitis. Hyp significantly reduced the levels of TNF-α, IL-6 and COX-2 and increased the levels of IL-10. Thus, Hyp exerts its preventative and therapeutic effects by correcting the imbalance between proinflammatory and anti-inflammatory mediators.

Increased rates of colonic apoptosis have been reported in patients with IBD and experimental colitis [33, 34]. Increases in the rate of intestinal epithelial cell apoptosis destroy the structure of the epithelial barrier and contribute to the development of intestinal damage [4]. B-cell lymphoma-2 family members, including Bcl-2 and Bax, play a vital role in cell apoptosis. Bcl-2 is an anti-apoptotic protein, whereas Bax is regarded as a pro-apoptotic protein, as it binds to and antagonizes the effects of Bcl-2 [4]. Increases in the Bax/Bcl-2 ratio increase the level of cytochrome c in the cytoplasm by inducing its release from the mitochondria. Cytochrome c activates Caspase-3, one of the major executioner caspases in the apoptosis pathway [4, 33]. According to our data, Hyp upregulates the expression of Bcl-2 and downregulates the expression of the pro-apoptotic protein Bax and Caspase-3, indicating that Hyp mitigates colonic apoptosis in the mouse model of DSS-induced colitis. These findings are consistent with those of a report on the inhibitory effects of Hyp on apoptosis in H_2_O_2_-treated human umbilical vein endothelial cells but are inconsistent with those of a report showing that Hyp enhances apoptosis in pancreatic cancer [14]. As shown in the study by Crespo I et al*.* [33], oxidative stress triggers the expression of several genes linked to apoptotic cell death. The effects of Hyp on colonic apoptosis are likely attributed to its ability to suppress oxidative stress, as epithelial cell apoptosis is enhanced when the intestinal mucosa is exposed to excess ROS levels under inflammatory conditions [35].

Therefore, we assessed the effects of Hyp on the Nrf2 signaling pathway and NF-κB p65 to elucidate the mechanisms underlying the anti-inflammatory and anti-apoptotic effects of Hyp. NF-κB, which plays a vital role in changes in the inflammatory phenotype, triggers the expression of several proinflammatory genes and enhances colonic mucosal inflammation in response to stress [1]. Colonic inflammation is regulated by the NF-κB pathway [25]. Based on our results, Hyp attenuates the inflammatory response by suppressing NF-κB activation, consistent with previous studies [30]. Nrf2 is a transcription factor linked to the transactivation of various detoxifying enzymes, and the Nrf2 pathway is known to regulate oxidative stress and the inflammatory response [12]. Kensler [8] confirmed that Nrf2 gene-knockout mice tend to be more susceptible to azoxymethane (AOM)/DSS-induced colitis than wild-type mice due to increases in the levels of inflammatory mediators and reductions in the levels of phase II detoxifying/antioxidant enzymes. Moreover, LPS-induced NF-κB activation is inhibited by Nrf2 activation [36]. HO-1, the main antioxidant molecule regulated by Nrf2, plays a critical role in inhibiting oxidative stress processes. The Nrf2 signaling pathway was recently reported to be involved in apoptosis. Activation of the Nrf2/HO-1 pathway attenuates apoptosis by inhibiting Caspase-8 activation [37], and Nrf2 activation plays a positive role in inducing the expression of anti-apoptosis genes, such as Bcl2 and Bcl-xl [38]. Uncontrolled ROS surges are a key trigger of mitochondrial dysfunction and mitochondrial breakdown, changes that eventually lead to cell apoptosis. In the present study, Nrf2, a major antioxidant transcription factor, and its downstream antioxidant enzymes, HO-1 and SOD, were activated by Hyp. The protective effects of Hyp may be mediated by the activation of the Nrf2 signaling pathway. The major limitation of this study was its lack of evidence supporting the hypothesis that Hyp exerts its beneficial effects by activating the Nrf2 signaling pathway. Therefore, in future studies, we will block the Nrf2 pathway to test this hypothesis.

## Conclusions

Based on the results of the present study, Hyp exerts protective effects on DSS-induced colitis in mice, effects that may be due to the suppression of inflammation and apoptosis via the activation of the Nrf2 signaling pathway, which exerts antioxidant effects.

## Abbreviations

ARE: Antioxidant response element
CMC: Carboxymethyl cellulose
COX-2: Cyclooxygenase-2
DAB: Diaminobenzidine
DAI: Disease activity index
DSS: Dextran sodium sulfate
HO-1: Hemeoxygenase-1
Hyp: Hyperoside
IBD: Inflammatory bowel diseases
Keap1: Kelch-like ECH-associated protein1
MDA: Malondialdehyde
Nrf2: Nuclear factor-erythroid 2-related factor 2
PVDF: Polyvinylidene difluoride
ROS: Reactive oxygen species
SOD: Superoxide dismutase
TBA: Thiobarbituric acid
UC: Ulcerative colitis
